# 

**Published:** 1887-02-26

**Authors:** 


### THE HOSPITAL

An Institution, Family, and Parochial Journal of Hospitals, Asylums, & all Agencies for the Care of the Sick, Criticism & News.

### CONTENTS.

**Faith-Healing** - - - - - - - - - 361
**Words of Consolation.**—XXII. Nurses' Trials—No Drudgery - - - - - - - - - 362
**The Coster's Customer: or, How I Earned Twenty Pounds.** By An Amateur Butler - 363
**Heart Disease: a Word of Comfort** - - - 365
**An Ambulance at Work** - - - - - 366
**Notes and News.**—Royalty and the Hospitals.—Miscellaneous Items.—Hospital and Infirmary Balls.—The Charing Cross Hospital.—The Essex Convalescent Home.—Lord Halsbury and the National Hospital for Paralysed and Epileptic.—The Beau Site Convalescent Home.—Vacancies.—Hospitals in India.—An Unexpected Donation.—The Cancer Hospital.—Donations and Legacies to Medical Charities.—The Bethnal Green Working Men's Society.—Hospital Saturday Fund: Meeting of Delegates.—The Worcester Ophthalmic Hospital.—The Peterborough Infirmary.—Jubilee Hospital Proposals, etc. - - - - - 368
**Annotations.**—Provident Dispensaries.—Aurard's Incubator.—Town *v.* Country - - - - - 371
**Reminiscences of the Insane.** By A Mental Physician - - - - - - - - - 372
**Nursing Institutions and Hospitals.** By Dr. J. C. Steele, Mr. Burdett, Dr. Bedford Fenwick, and Dr. G. W. Potter - - - - - - - 373
**The Editor's Letter Box.**—Uniformity in Hospital Accounts.—Nurses' Pension Funds - - - - 375
**A Retrospect of Christmas** - - - - - 376
**The Children's Corner.**—Puzzles. Answers, etc. - 377
**Amusements with Profit** - - - - - 377
**In- and Out-Patients' Hospital Diary** - - - 378

